# The intentionality of smell

**DOI:** 10.3389/fpsyg.2014.00436

**Published:** 2014-05-27

**Authors:** William G. Lycan

**Affiliations:** ^1^Department of Philosophy, University of North CarolinaChapel Hill, NC, USA; ^2^Department of Philosophy, University of ConnecticutStorrs, CT, USA

**Keywords:** smell, intentionality, representation, olfaction, objects

## Abstract

If any sense modality represents, vision does, but argument is needed to show that smell does. This paper rebuts two reasons for doubting that smell represents, and offers several arguments that it does. The paper then considers several recent proposals as to exactly what a smell represents, and defends a version of the author's original proposal—that a smell represents a miasma in the air—against its competitors.

If any sense modality represents the world, vision does. But argument is needed to show that smell does; it has never been obvious that smell represents. This paper rebuts two reasons for doubting that smell represents, and offers several arguments that it does. The paper then considers several recent proposals as to exactly what a smell represents, and defends a version of my older proposal—that a smell represents a miasma in the air—against its competitors, though offering a concession or two.

In the next section I shall expound the main reasons for which people have denied representational status to smell. But first, I must say more precisely which of several possible things I mean by “smell.” The word could mean, (i) olfactory *experience* as some writers put the question, (ii) *smelling*, whether “experienced” (i.e., consciously) or not^1^, or (iii) the olfactory *system* as investigated by cognitive and neuroscientists. I shall try to mean (ii). (iii) Would be an entirely empirical matter, though obviously scientific results regarding the olfactory system bear fairly directly on our own issue. Moreover, it is possible that the olfactory system subpersonally represents properties that are not smelled by the whole person whose olfactory system it is. (i) seems needlessly restrictive, since (according to me) the difference between a type of mental state occurring non-consciously and that same type of state occurring consciously is superficial, a matter of whether the state is itself represented by a higher-order state (Lycan, 1996, 1998, 2004); it would not normally affect the state's own representational content.

So my opening question more precisely is, are worldly properties or things represented in and/or by person-level smelling? And as noted, I begin with the negative view.

## The case against

(1) There are two main reasons for doubting that smell (in particular) represents. First, if we focus introspectively on the specifically sensory character of an olfactory experience, we detect only that modification of our consciousness, the qualitative condition or event in us. Even if we infer the presence of horses from their characteristic smell, the smell does not itself present horses as its own representatum. We infer horses only because we already know from experience that that smell is typically produced by horses. (The overall phenomenology perhaps suggests otherwise: we just smell horses and notice no inference. But again, the smell has to have been associated empirically with horses; if you experienced it having never made that association, it would say nothing to you^2^.)
  Second, were you to experience a smell that represents X when no X is present, you would be misrepresenting, smelling falsely or incorrectly. But this does not seem to happen. There used to be a distinctive way in which a new American car smelled from the inside. Then someone manufactured an aerosol that replicated the new-car smell. You could buy it in auto parts stores; I think it was actually called “new-car smell.” Suppose a friend had used the fake and given you a ride. The car smelled new to you even though it was several years old. This is misrepresenting of some sort, but it is not incorrect smelling. The nose itself was *not fooled*, for the new-car odor was really there and causing the smell experience in the normal way; the car's inside did have the new-car smell even though it is not a new car.
  We characterize smells by reference to their normal environmental causes: horses, new cars; the smells of roses, natural gas^3^, boiled spinach, locker rooms. But it does not follow that those causes are represented by the smells, because they can be counterfeited by other substances. Again, the point is not that we can misrepresent them; that would hardly show that they are not representata. Rather, in a counterfeit case the nose is not fooled, and the smell itself is not misrepresenting, even though we may form a false belief; and nothing yet has shown that we smell correctly either. (Conversely, of course, if you experience the locker room smell when you are not in a locker room, you are not smelling correctly, but it does not follow that you are smelling incorrectly ^4^.)
  Nor do phenomenal smells represent, as some Gibsonians would have it, broader ecologically significant properties of things, for the same reason and for others^5^. Thus, it is tempting to conclude that smells merely accompany external objects with a fair degree of type–type reliability, but do not represent them.

## Rebuttal

(2) That is the case against smell's representing. It is not a very strong case. Vs. the first argument: Though introspection does seem to reveal that vision represents, its failure to do that for smell hardly shows that smell does not represent. Introspection is a blunt instrument, and cannot in general be relied on to determine whether a mental state has representational content. Witness the fact that philosophers disagree on such questions, despite being roughly equal in introspective competence. Further, though some mental states traditionally have not been thought to represent, there has been increasing consensus that those states do have some representational structure even though that is not functionally the most important thing about them and, more to the point, even though their representational structure does not leap out in introspection: pain; some of the emotions^6^.
  The second argument fails because there is no reason to assume that if smells represent at all, they represent the environmental causes by which we casually characterize them. As we shall see, there are several other candidates for representata. (I say no reason to *assume*; this is not to deny that a theory of representation^7^ for smell might invoke such causes, though the second argument would itself count against such a theory^8^.) And now I shall argue positively that smell does represent.

## The thesis

(3) Consider that a main function of any sense modality is feature detection, the registering of environmental properties. It does not follow that the relevant sensory states represent those properties—at least not without addition of the dubious premise “If a state has the function of detecting feature F, the state represents F”—but I and others have argued positively that smell does represent. I claim that a smell actually has semantical properties: reference, a truth- and/or satisfaction-condition. A smell can be treated formally, *ála* Hintikka, as a function from possible worlds to truth-values, and any such function corresponds to a proposition expressed^9^. A smell can be incorrect, a *mis*representation. If these perhaps surprising things are true, then surely smells are indeed representations.
  And I believe they are true. It may *seem* that, phenomenally speaking, a smell is just a modification of our consciousness, a qualitative condition in us, lingering uselessly in the mind without representing anything. And as noted, disinclination to think of smells as representations increases when we ask what they might be representations of. If the “smell of roses” represented roses, then it would be true or satisfied or correctly tokened only in response to roses, false or incorrect otherwise; and it would determine a function that, given a world, spit out exactly the set of roses at that world. The rose smell does neither of those things. Yet, as I have observed, there are other candidates for external representatum.
  For one, consider what an *odor* is, in one public sense of the term. It is a miasma in the air, a vaporous emanation, a diffusing collection of molecules typically given off from a definite physical source. It is itself a determinate physical thing, distinct from its source object, that makes physical contact with the smell receptors in one's olfactory epithelium and sets them to firing. We are publicly, commonsensically and often mutually aware of such odors; they are public physical entities available for sensing by anyone who happens by. Now, an odor is a candidate for representatum, and the idea of an odor as an intentional object of smell resists the objection I have made to the more colloquial candidates. For things other than roses can give off the odor “of roses,” and roses can fail to give off that odor. (Again, I am talking only about the match in the physical world between types of object and types of odor.) Perhaps, then, smells represent odors^10^.
  But why think that, even so? Perhaps, to the contrary, all there is to the relation is that smells are highly but imperfectly correlated with odors, and that is not enough to make for a case of representing.

## The case for

(4) Actually there are several positive arguments for awarding representational status to smell, now that the main objections have been circumvented. A first is that once smells are correlated with odors rather than with types of object, a kind of incorrectness does manifest itself, hence a correctness- or truth-condition. If I hallucinate a rose smell in the absence of any rose *or anything else* that is giving off the rose odor, I am misperceiving. The point is not just that my belief that a rose is present is erroneous. I may not even have that belief, knowing full well that my olfactory experience is hallucinatory. Something is perceptually wrong; my olfactory bulb is saying “Rose odor” when there is no rose *odor* physically present, and that report is a lie. Where there is falsehood there is representation.
  [Of course this can be resisted. One can grant that a detector or indicator is registering a false positive without being forced to admit full-bore representation, if one wishes to place further conditions on what it takes for something to be a genuine representation (cf. Ramsey, 2007)]. My claim for this first argument is only that smell has a possibly unreal, non-actual representatum in at least the rudimentary sense that detectors and indicators have representata. However, I would add that smell's strong and multifarious functional connections to memory and other cognitive agencies suggest a stronger representational connection as well.]
(5) Richardson (2013) persuasively attacks the phenomenological view on which our first anti-representationalist argument was based (section 1 above), that “considered only phenomenologically, a smell seems a modification of our own consciousness rather than a property of a perceptual object that would exist unperceived” (p. 406, quoted from Lycan, 2000, p. 277). She argues to the contrary that smell, like vision, is “exteroceptive,” i.e., that even phenomenologically, the sensible qualities inhering in a smell sensation “seem … to be qualities of objects distinct from our bodies” (p. 405), the “objects” for the case of smell being odors, and the seeming is not just a matter of cognitive association^11^. Exteroceptivity is also connected to *finding out*: “In exteroceptive experience we find out about the [relevant] qualities of objects … by their seeming to have the qualities in question” (p. 406). Richardson carefully makes the case that olfactory experience is exteroceptive *despite* its lack of vision-like spatial features. I am persuaded that she is right.
  N.b., exteroceptivity does not entail representation; it is phenomenological only. (Nor does Richardson claim that smell represents.) But this gap can be bridged using a type of argument deployed by Byrne (2001) on behalf of the Representational theory of sensory qualities (see again note 9; cf. also Thau, 2002). He appeals precisely to the notion of seeming: “if the way the world seems to [a subject] hasn't changed, then it can't be that the phenomenal character of his experience *has* changed” (p. 207). Suppose a subject has two consecutive experiences that differ in phenomenal character, i.e., in how they subjectively feel to her. If she notices the change in phenomenal character, Byrne argues, the way things seem to her when she has the second experience must differ from the way they seemed to her while she was having the first. For suppose that consecutive experiences are the same in content. Then the world seems exactly the same to the subject during both. She “has no basis for” noticing a change in phenomenal character either, and by the previous premise it follows that there was no change in phenomenal character (p. 211). Byrne concludes that experiences cannot differ in phenomenal character without differing in representational content^12^.
  Thus, if a subject's phenomenology changes merely by the addition of an olfactory component, there must have been a change in representational content, and the obvious candidate is the addition of an olfactory representatum.
(6) A further argument can be adapted to the purpose from one of Moreland Perkins' (1983, p. 63ff). Perkins points out that when we sniff an object and for the first time perceive its odor, we find out something about the object. What we find out is, seemingly, its odor. Now according to the view I have expounded earlier, odor is just a physical diffusion of relevantly shaped particles in the air. But, Perkins argues, what I find out when I “find out the object's odor” is not (*per se*) anything about a physical diffusion of relevantly shaped particles in the air. Rather, to find out the odor in the relevant sense is to find out what the odor is like to smell.
  “Like” in that last formula, as in the phrase “smells like,” does not mean resemblance. In Perkins' Farrell-Nagelian sense, one can find out and know what a new odor smells like without there being anything in one's previous experience that it resembles. Perhaps we would do better to speak of finding out how the odor smells; “It smells like this” and “It smells this way” do not seem to differ in meaning. How the odor smells is something that one can know only if one has either actually smelled it or has smelled something sufficiently similar that one can be told by comparison how the first odor smells.
  I shall break off at this point in Perkins' line of reasoning (which is actually a long defense of a version of a none too clear doctrine he calls “Indirect Realism”), for it is all I need as a foundation for my own argument. Much to the alarm of all, however, I shall start off in my own direction by noting that one could know all the objective scientific facts there are—about a physical odor, its effect on the olfactory epithelium, the ensuing excitations in the bulbs, and all further results in the thalamus, the neocortex, the limbic system and so on at any length—without knowing what the odor smelled like (how it smelled).—Please, stay calm. I am not about to invoke Leibniz' Law and infer that there is a fact left out which eludes all of science and which philosophical materialists feloniously ignore. Not even Leibniz' God would have the power to make that follow, for it simply does not follow^13^. However, we do have to make sense of the admittedly odd fact that I can know that P and fail to know that Q even when the fact that P just is the fact that Q on a suitably coarse-grained individuation policy for “facts.”^14^
  I can know that I have salted a tomato without knowing that I have put NaCl on it, even though the fact of my salting it is one and the same as the fact of my putting NaCl on it. That is because what is the very same substance, salt or NaCl, can be represented by me in each of two psychologically inequivalent ways. Knowing is hyperintensional; if you like, its object is not just a fact but a fact under a particular representation.
  As is painfully well-known, this hyperintensionality is similarly manifested by mind-brain identities. Suppose for rude simplicity that pain is simply the firing of X-fibers. Even if that were so, I could know that I was in pain without knowing anything about X-fibers, and I could know physiologically all about X-fiber firings without knowing what it is like to feel pain, if I have never felt it myself (had my own X-fibers firing). This is possible, again, so long as the same fact is represented in two psychologically inequivalent ways. The fact, of the pain or (indifferently) the firings, can be represented in a public way, in physiological terms, as well as by the use of the English word “pain.” But much more commonly it is represented introspectively by its owner—for short, it is felt. One can know all the publicly accessible facts about pain without knowing what pain feels like so long as one has not introspected any pain oneself. One comes to learn what it feels like when one first does introspect it, when one thus begins to represent it in a first-person way.
  So with smell. I can know all the chemistry of a rose, the physical properties of the rose odor, the neurophysiology etc. of olfaction and all the other scientific facts about smell without knowing how the rose odor smells. But how the rose odor smells, or what it smells like, just is the complex of fact I have just mentioned, which by hypothesis I do know. The appearance of contradiction can be resolved just as in the case of pain, and (more to the point for my argument) I do not see any other way of resolving it: I can know the complex of osphresiological fact without knowing how the rose smells because knowing is knowing-under-a-representation, and the same fact can be known under one representation but not under another. Here, I know the facts under their textbook descriptions; what I fail to do until I have smelt a rose is to know them under their introspective descriptions, their first-person representations supplied by my introspector. I will come to know what the rose odor smells like only when I do represent it introspectively, that is, when I smell it. That is how the Farrell-Nagel puzzle is resolved. And that solution entails that olfactory experience involves representation.
  However, a gap remains: I have so far ignored a complication needed to make the smell story truly parallel to the case of pain. There are really two tiers of representation. The rose odor causes an olfactory sensation, a smell, which represents it, and that sensation is in turn represented by my introspector—an attention mechanism—when I concentrate on the sensation's phenomenal character. (If I never do attend to the smell-sensation, if my introspector happens never to be directed upon it, then I will never be consciously aware of the sensation; cf. unfelt pain. This picture is defended at length in Lycan, 1996). Now, someone might ask, though the present Nagelian argument does require that the introspector's output be a representation, why should we believe that its representatum, the first-order smell sensation, itself represents the physical odor? Perhaps it is merely caused by the odor before being represented by the introspector. I reply, again following Perkins, that according to common sense and parlance, what we come to know when we attend to the smell of our first rose is what the rose odor smells like (and derivatively what the rose itself smells like), not just what it feels like to have the smell sensation that happens to have resulted from contact with the odor; as Perkins said, we find out something about the odor, viz. how it smells. I see no reason not to take common parlance at face value here, and accept that the smell sensation ascribes a sensory quality to the odor itself ^15^.
(7) The picture I have presented yields an explanation of a further phenomenon: the ineffability of smell, the fact that smells (and odors) can be described in words only by comparison to other smells (and odors) or by reference to their external causes. For my internal representation of an odor is a lexeme of a private language, the medium of representation in which my introspector makes its reports. That lexeme has any number of co-referring descriptions framed in public natural languages, but (for reasons emphasized in Lycan, 1996) no such description that shares its meaning. Nothing equivalent can be said in English (though of course we could introduce a public word—“samantha”—and try contra Wittgenstein to stipulate that it is to be synonymous with my introspective term^16^.
  Recently it has been suggested that the ineffability thesis has been exaggerated and that cross-cultural data refute or at least impugn it. Majid and Burenhult (2014)^17^ investigated a claim they express variously: that “the experience of a smell is impossible to put into words” (abstract, p. 266); that “people find it difficult, if not impossible, to name odors” (p. 266); that “people universally struggle to describe odors” (p. 269). These things may be true of Anglo-Americans and others from “Western Educated Industrialized Rich Democratic communities” (p. 267), but they are not true of some less urban and industrialized peoples. In particular, Majid and Burenhult studied the Jahai, a nomadic hunter-gatherer tribe on the Malaysian peninsula, and found that they have a very rich lexicon of odor terms, comparable to their color vocabulary.
  But those results do not bear on my own still pretty traditional ineffability thesis. The claim is not that we cannot name or identify or classify odors—though we Anglo-Americans cannot as well as the Jahai do. Majid and Burenhult's first formulation comes closest to it (“the experience of a smell is impossible to put into words”), the operative term being “experience”: The claim is that once we do have a subjective smell all named and identified and classified, *then* if we are asked “But what is it like *in itself* to experience that smell [so named, identified and classified],” we go tongue-tied.
  Doubtless the foregoing case for smell's representational status could be resisted by a sufficiently determined anti-representationalist, and we know they are out there. But I believe it is a strong case, and, importantly, it militates against *selective* anti-representationalism. That is, it makes it hard for a theorist to admit that vision and perhaps hearing represent but deny that smell does.
  I return to the question of *what* smell represents. My position to date has been that it represents odors in the sense of particulate miasmas in the air, but in recent years differing proposals have been offered.

## What does it represent?

(8) Vision *ostensibly* represents things or objects and ascribes properties to those things. Batty (2010) asks whether an olfactory experience does the same. Introspection cannot settle that question. Instead, Batty first appeals to the fact that there are no known olfactory illusions (as opposed to hallucinations), i.e., cases in which a real object is perceived as having a property that it does not have^18^. Second, she observes that there is no obvious Many-Property Problem (Jackson, 1977) for smell^19^. Third, she argues that smell *per se* is not spatial, and so cannot (alone) identify a worldly object spatially; nor is there any other means of doing so^20^.
  On their face, these points would suggest that smell does not represent in the first place. It may once again now seem that “olfactory experiences are mere smudges on our consciousness” (p. 518). But Batty does not accept the points as showing that, for as she rightly says, having representational content does not entail representing particulars and ascribing properties to them^21^. Rather, she suggests that smell represents properties “abstractly,” as merely existentially quantified: *There is F-ness here* (where “here” means only something like, present to me^22^.) What in fact makes such a quantificational content true is something in the air—something we know on other grounds to be an odor—but that is itself no part of the bare representational content.
  I am not sure how much disagreement there is between Batty's “abstract” theory and my (1996) view. The latter certainly did not entail that smell represents odor particulars in the way that vision (allegedly) can. It does entail that smell represents odor universals. Disagreement depends on detail. As her leading example (p. 530), Batty offers “∃*x*(*x* is smoky, lavendery & at L_0_,” where L_0_ is the default ambient location for all smells and “smoky” and “lavendery” are counterparts of the color and shape predicates that would figure in a parallel “abstract” visual representation.
  If “smoky” and “lavendery” are being used as adjectives, then as Batty seems to intend, the semantics is blind to *x*; *x* is an I-know-not-what that nonetheless smokyizes and lavenderizes. But if the “is” is that of instancehood, “smoky” and “lavendery” can be taken as kind terms, and the representation can be read as, “There is some smoky and some lavendery at L_0_.” (We know, as the natural-kind semantics does not, that smoky and lavendery are really odors, i.e., miasmas in the air.) It is this second interpretation that is hard to distinguish from my view.
  The adjectival interpretation invites the question of what properties smokiness and lavenderiness are, given that they are properties of odors rather than odors themselves. Perhaps they are response-dependent relations between odors and perceivers. But this would raise problems of the sort that bedevil dispositional theories of color, and Batty has not (at least not here) supplied motivation for doing so.
  The natural-kind interpretation forces a question for Batty about entailment: Does “There is some smoky and some lavendery at L_0_” entail both “There is some smoky at L_0_” and “There is some lavendery at L_0_”? If it does, I see little difference between her view and mine. If it does not, we need to hear more about how to parse her formula, “∃*x*(*x* is smoky, lavendery & at L_0_.” What *would* be the relation between “smoky, lavendery,” “smoky,” and “lavendery?”
(9) Young (2013) challenges the odor theory, at least a strong and distinctive form of it. His main complaint is that existing odor theories have either implied identity and individuation conditions for smells, which conditions are untenable, or they have been sullenly silent on identity and individuation and therefore need filling out by tenable conditions. On the first horn of that dilemma, his paradigm seems to be the Platonic idea that an odor is an emanation of “detached proper parts of ordinary objects” (p. 3). That idea explains some of the phenomena noted in section 1 above, such as the veridicality of a smell even though the source object is itself long gone. But, Young argues persuasively, smells do not always track their source objects or even (at the level of types) their usual or typical source objects.
  Of course, odor-theory phrases like “a miasma in the air” do not commit us odor theorists to the Platonic source-object thesis, but that is because they are vague. Young demands that we be able to tell him *what types* of miasmata, exactly, produce which smells. Why, for example, does a particular synthetic chemical compound have the same olfactory quality as a rose (p. 7)? He offers a proposal that he defends by appeal to a good deal of empirical literature. (He is willing to call the proposal a “modulation of” the odor theory.) The proposal is to identify olfactory objects, not with molecular compounds, but with “the three-dimensional chemical structures of molecules” (p. 21), “actual three-dimensional structures formed by their constituent functional groups and their placement in space and time.”
  If I understand Young correctly, the main contrast between this Molecular Structure theory and “the Odor theory” (now capitalized) is that it appeals to “micro-objects derived from the structure of matter” rather than to merely small bits of a source object (p. 27). If “the Odor theory” is thus characterized, I believe Young wins and anyone who holds it should stop doing so. But I know of no one who does hold it as such; I and the other odor theorists whom I have read fall on the other horn of his dilemma, simply not having said enough about the relevant “miasma” to be tested against the science.
  Nor am I nearly knowledgeable enough to contest his assessment of the empirical findings. If the operative elements of what hangs in the air are situated three-dimensional molecular structures, so be it, and I provisionally accept the friendly precisification. But I have one comparatively a priori worry about it.
  Young grants (section 4.2) that a synthetic odorant that mimics the smell of a natural object may differ from that object in molecular structure. Does this not create a problem of metamers? For predictive purposes, he need furnish only bottom-up sufficiencies, and his theory will succeed by his lights if he is able to predict that structure A will trigger a rose smell, and so will structure B and so will structure C. But our own question was, exactly what does the “rose smell” represent? In the face of metamers, is it now ambiguous as between three different representata, A, B, and C (and however many more synthetics the future might reveal), so that it will differ contextually in accuracy condition?
  It is tempting to look for a more abstract, higher-level property that is being implemented by A, B, and C; but Young explicitly rejects that move (p. 27). Yet the ambiguity view is an extreme form of externalism, and though generally an externalist myself, I do not think perceptual representation in particular should be so fraught. As a friendly retreat from Young's friendly precisification, I offer the analog of my own view of color metamers (Lycan (1996); not that I think Young would welcome it)^23^.
  The view construes color representata as physical properties of objects, but only as very modest ones. They are roughly the properties that constitute the objects' dispositions to produce the corresponding sensations in normal sentient observers under normal viewing conditions; and as we all know, those properties are an unruly, rough, and ragged lot. Certainly they form no natural kind at the level of physics or chemistry. A physical color, then, is taken to be a pathetically disjunctive microstructural property of objects. It is of interest only because of its relation to the human visual system.
  The latter fact seems damning: “(1) You pick out the ‘property’ in question only by reference to the human visual system. And in fact, (2) all ‘its’ instances have in common is that they do produce the relevant sensations in people. Moreover (3) you have admitted that it constitutes its subject's disposition to produce such sensations. For these reasons, the property you're talking about is just that of *being disposed to cause people to sense* in the corresponding way.” We had been trying to explicate phenomenal color as a matter of representing worldly color, but now we are tacitly understanding “worldly color” in terms of the phenomenal; circular and viciously so.
  But the foregoing argument is a bad one. As admitted, the kind of property I am talking about is ontologically and scientifically ugly; but not even the conjunction of the premises (1)–(3) justifies the argument's conclusion. Despite its ugliness, my sort of property inheres in an object on its own; regardless of how it is picked out or identified by me or anyone else, regardless of its ever producing sensations in anyone (or being detected by any being at all), and, surprisingly, regardless of its actually constituting a disposition to produce sensations in anything. For in principle it can be specified or defined independently of its doing any of those things. It is as it is, whether or not anyone identifies it or refers to it, whether or not it ever produces sensations of any sort, whether or not it constitutes any disposition, and even if none of those things were true.
  If this view, unsatisfying as may be, is nonetheless correct, it can be extrapolated to the problem of smell metamers. What a smell represents is then a (probably open-ended) disjunctive property—the property that serves as the categorical basis of an odor's being disposed to cause the corresponding smell sensations in humans, but not (metaphysically) because it so serves. This removes the threat of multiply ambiguous reference, at the cost of positing a single referent that is in its own metaphysical right ugly and misshapen.

## Layering?

(10) One may tentatively suppose, then, that phenomenal smells represent odors in the sense I have tried to specify. But (in conversation) Ruth Millikan has contended against me that my arguments and others' against the idea that smells represent environmental objects must be flawed. If we are to agree with anything like her pleasantly Panglossian evolutionary-historical account of representational content (Millikan, 1989, 1995), we must suppose that if smells do represent anything, they do after all represent environmental objects of potential adaptive significance. Surely that is what olfaction is *for*, to signal food, predators, shelter, mates, and other objects of interest ultimately derivative from those; and signaling is at least a crude form of representing.
  I am inclined to think Millikan is right about that (bar “if smells do represent anything”). And there is further pressure to expand the field of representata and make it still more distal: Phenomenologically, it seems that we do smell roses, horses, and even individual things or people.
  But these two considerations do not force me to abandon my claim that smells represent odors. Even if I accept them both, I suggest that smells represent adaptively significant environmental entities, and worldly things and people, and they *also* represent odors. In fact, they represent the environmental entities *by* representing odors. By smelling a certain familiar odor I also smell—veridically or not—an actual rose or roasting lamb or my least favorite aunt.
  Let me back up. The present sort of issue has long since been encountered in the philosophy of vision^24^. In that literature, there are conservative positions (vision itself represents only colors and shapes), very liberal positions in the tradition of Hansen and Kuhn (suitably trained and informed vision can represent practically anything), intermediate positions (vision can represent natural kinds and causal relations, but not expensiveness or uninhabitedness or uninhibitedness or global warming or an economic downturn)—and layering positions, according to which visual states have multiple intentional objects and we see more abstract and worldly things in and by seeing simpler and more primitive ones.
  Here are some of the layering views. Peacocke (1992): We represent indexical “scenario” content; low-level properties (non-conceptual); and high-level properties; it is, I think Peacocke meant, the same vehicle that does both representings. Lycan (1996): We represent high-level properties by representing scenario content and low-level properties of external objects. The model here is that of deferred ostension: Pointing at a chalk mark on a blackboard, we refer to a numeral; thereby we refer to a number; thereby we refer to an office in Emerson Hall; and thereby we refer to its occupant, a person. Noë (2004): We perceive high-level properties, though only as “present as absent,” by actually-perceiving “perspectival properties” (=“appearance properties”) of external objects. Schellenberg (2008): We perceive “situation-dependent” properties of external objects, and thereby the high-level properties of the same objects, the perception of the latter depending epistemically on that of the former. (It is important to grasp that the situation-dependent features are perfectly real and mind-independent.)
  Returning to my original argument against the idea that the rose smell represents roses: It was essentially that if I experience the rose smell when the rose odor is present but no actual rose is, I am smelling correctly, and if I experience the smell when an odorless rose is present, I am not smelling correctly. But in the face of my own layering view for the case of vision, this argument is too simple. For that view introduces the possibility that a mental representation can have more than one truth value at once. And indeed, I think it is fairly plausible to say that in the first case—that of experiencing the rose smell in the absence of any rose—I am representing *both correctly and incorrectly*, the odor correctly and roses incorrectly.
  My suggestion, then, is that a given sensory state typically has, not just a single intentional object, but two or more arranged hierarchically by the “by” relation. As before, a good model here is that of deferred linguistic reference.
  But there are problems. The first is the above-noted objection that my rose representation is only the result of unconscious inference from the lower-layered olfactory representation rather than being olfactory itself, especially since the “rose” response seems to depend on acquired empirical association. That objection is not decisive, but it is serious^25^.
  My (1996) layering view depended on a visual ontology of “shapes,” some of which items are real physical objects but most are non-actual. Whether or not one can abide that ontology, it has no obvious analog for smell, because the notion of a “shape” was motivated by visuocentric considerations of size, direction, distance, and surface. I have given up my (1996) position in favor of Schellenberg's superior layering view. But (second problem) it is far from obvious that her “situation-dependent” properties have olfactory analogs, either. Situation-dependent properties are, she says, “(nonconstant) functions of the intrinsic properties of the object and the situational features” (p. 60). The cup on the table has
  one side closer [to me] than the other; one part faces away from me. Its shape is presented in an egocentric frame of reference, which in turn means that the object and its parts are presented as standing in specific spatial relations to me. The way the cup is presented to a location is on the suggested view an external and mind-independent, albeit situation-dependent property of the world. Any perceiver occupying the same location would, ceteris paribus, be presented with the cup in the very same way. (p. 61)
  Schellenberg says similar things about color: The relational properties of an external object that make it appear colored to us as it does in this setting and lighting conditions are, tautologously, properties of the object.
  Do objects have mind-independent, situation-dependent odor properties? Obviously roses have relational properties which cause them to smell as they do to us. But I do not offhand see that simply by detecting those properties we detect roses without benefit of background knowledge. Perhaps I am wrong.
(11) I believe that I together with others have made it very plausible that smell represents, and I have defended roughly my original view of what it represents first and foremost. I would still like to accommodate Millikan and common parlance in the matter of ecodistality, through layering. But I of all people cannot assume that a view that is plausible for vision will extrapolate to any other sense modality.

### Conflict of interest statement

The author declares that the research was conducted in the absence of any commercial or financial relationships that could be construed as a potential conflict of interest.

## Footnotes

^1^Some writers use “experience” liberally, to include all cases of perceiving whether conscious or not. I prefer to reserve the term for sensing consciously, i.e., for sensings of which their subjects are aware.

^2^“Think, when we talk of horses, that you see them, printing their proud hoofs i' the receiving earth.” Despite the point about empirical association, a more immediate way to imagine them is through their smell. An anonymous reviewer has pointed out that vision too requires empirical association. If I am acquainted with horses only through the sounds they make, but then for the first time I come upon a field containing horses, cows, and ostriches, vision will not tell me which of the animals are horses. It is controversial whether vision ever represents natural kinds. Siegel (2010) argues that it does; for discussion, see Lycan (2014). It is not very controversial that vision does represent, but there are a few opponents, e.g., Campbell (2002).

^3^Such characterizations can be tricky in particular cases. Natural gas itself consists largely of methane (CH_4_), and is odorless. What we commonly call the smell “of gas” is actually that of a pungent odorant added by the gas company in order that an otherwise fatal gas leak will be readily apparent; the connection between the adulterant and the gas itself is entirely conventional. So the smell “of gas” is not that of gas, but of the chemically unrelated odorant.

^4^The point holds even of smells that purport to identify individual things, such as the competent dog's master-indicating smell. Although for a dog the smell of an individual human being may be unique in fact, it is still true that were another human being to duplicate the dog's master's particular smell, and the dog were to smell that smell and expect master, the dog would not be mistaken in smelling it, on something else that did smell the same way, even though the dog's ensuing expectation would be false. Here too (cf. fn 2, and thanks to the same reviewer) there is a visual analog. The interior of an old car may have been freshly painted and detailed, causing you to believe that the car is new, but the eyes themselves are not fooled. It seems to me very unlikely that vision represents so sophisticated a category as “new car” (on this, see again Lycan, 2014). But here too, it is not widely doubted that vision does represent.

^5^See Ch. 3 of Perkins (1983).

^6^Pain: Armstrong (1968), Pitcher (1970), Tye (1995, 2005), Crane (2003), Hill (2005). Emotions: Prinz (2004). Lycan (1996) argues that even the vaguest moods, such as “free-floating” anxiety, depression, and general optimism, have definite though comparatively uninteresting representational content. Vivat Brentano.

^7^A “psychosemantics,” to the philosophers.

^8^In sec. 10 below I shall allow that the normal environmental causes may be represented by smell experiences, albeit in an indirect way.

^9^This is a standard account of the propositional meanings of natural-language sentences. E.g., “There is a moose in Caldwell Hall” is false in our actual world, but is true in some other possible worlds; the function from worlds to truth-values represents the range of circumstances under which that sentence would be true.

^10^If smells represent odors (or any other external phenomenon), it is open to one who holds the Representational theory of sensory qualities—“qualia” in one of that mutilated term's many senses—to identify those qualities inhering in smell experiences with the relevant representata. (According to the Representational theory more generally, any sensory quality such as a color, pitch, taste, or texture is, veridically or not, being represented as a feature of something in the environment. For example, if I have a yellow patch in my visual field occasioned by seeing a lemon, the mental yellowness is just that of the external lemon, represented by the visual system in the experience. If I hallucinate a similar lemon, the mental yellowness is still just that of the now non-actual external lemon, non-veridically represented in the experience.) I myself do hold the Representational theory and do so identify subjective smell qualities with properties of external odors (Lycan, 1987, 1996), but I also insist that the overall phenomenal character of a smell experience outruns the experience's representational content, because the specifically sensory quality in question is only a proper component of the overall phenomenology (Lycan, 1998). The main dialectical connection between the present paper and the Representational theory, then, is this: If I am wrong here and smell experiences do not represent, then either the Representational theory is false or, surprisingly, smell experiences do not feature sensory qualities at all.

^11^“This castle hath a pleasant seat. The air nimbly and sweetly recommends itself unto our gentle senses.”

^12^For the record, I reject that blanket conclusion; see footnote 10. But we can understand Byrne as using the term fairly narrowly, excluding, e.g., conative and affective features of the experience.

^13^That and why it does not follow has been explained at length by so many writers in philosophy of mind that it would be tedious even to begin naming them.

^14^One can of course hold out for a finer-grained individuation policy, as recommended by Chisholm (1976). But that policy proliferates “leftover facts” everywhere, not just in the philosophy of mind. On this issue in particular, see Lycan (2003).

^15^There is a perennial problem, much discussed in the “self-knowledge” literature, of how an introspective representation of a first-order mental state represents the latter state's own representatum in particular. But that problem is everyone's, or at least afflicts any view that posits higher-order representation of a state that is itself representational. It is not a special objection to the present argument. To pursue the analogy with pain: When I first felt the pain I now have in my left shoulder socket, did I find out anything about my shoulder? I believe so. I found out that there is damage or some other physical disorder in it—which belief was then confirmed by X-rays.

^16^For much more detail and defense of all this, see again Lycan (1996). Austen Clark (1993) speaks of a transparent/opaque ambiguity in phrases like “lilacs odor.” Taken transparently or de re, the latter phrase must refer to what some lilacs are up to, regardless of the smell sensation produced. On its opaque use, it describes the qualitative character of an olfactory experience regardless of what is going on in the environment.

^17^Thanks to a reviewer and to Bence Nanay for bringing this literature to my attention.

^18^Most philosophers have accepted this, but the psychologist R. J. Stevenson (2011) does not. He cites a number of empirical studies (cf. in which are shown mismatches between olfactory percepts and the stimuli that caused them. Batty (2014) does not dispute the data, but argues that such mismatches do not rise to the level of illusion intended by the philosophers.

^19^That problem was an objection raised by Frank Jackson against “adverbial” theories and others that tried to avoid commitment to individual phenomenal things' figuring in a subject's phenomenal field. A visual field, for example, cannot be described simply by means of a list of color properties and shape properties such as {blue, red, circle, triangle}, because that list alone would not distinguish a field containing a blue circle and a red triangle from one containing a blue triangle and a red circle. Even phenomenally, colors and shapes group into individual objects. Batty's point goes back to Clark (2000) and Smith (2002).

^20^In such arguments she follows Smith (2002) and Matthen (2005).

^21^Later in her paper (pp. 513–514) she does offer two quick arguments for the claim that smell represents, without supposing they are decisive: Each is an instance of what she calls the Unification Thesis: that “certain philosophical issues about perception should be settled in the same way for each of the sense modalities.” (1) All senses function as informational systems. “As guides of behavior and grounds of belief, the experiences of the sense modalities form a common kind.” (2) In particular, since some animals' olfactory experiences “are for them as vision is for us,” in that they are clearly “world-directed” and help the animals map the world, then if our visual experiences are representational, so are their olfactory experiences; and if their olfactory experiences represent, then so do ours however less richly.

^22^Richardson rightly urges that the location is more specific: present to my nose. This distinguishes odor from, e.g., ambient temperature, where temperature is perceived as being “around me” more generally.

^23^It is inspired by if not just swiped from Armstrong (1984, pp. 170–182; also 1987).

^24^For summaries, see Siegel (2010) and Lycan (2014).

^25^Philosophy has not entirely resolved the general question of whether the incredibly busy pre-processing that goes on in our perceptual modules should be counted as un- (because pre-)conscious inference—or how modular the modules are to begin with, given what is now called “cognitive penetrability” (Macpherson, 2012); on these matters, see Lycan (2014). For now: Even if my rose representation is the result of unconscious inference in one sense, that does not disprove the claim that it is also itself olfactory. A reviewer has further suggested that the mechanism connecting the rose odor to the rose is, rather, cross-modal binding.
